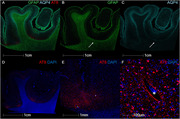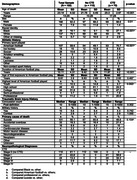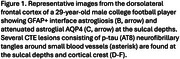# Young Amateur Contact Sport Athletes Are At Risk for Structural Brain Injuries and Chronic Traumatic Encephalopathy (CTE)

**DOI:** 10.1002/alz70855_105724

**Published:** 2025-12-24

**Authors:** Bobak Abdolmohammadi, Katharine J. Babcock, Jesse Mez, Michael L Alosco, Jonathan D Cherry, Madeline Uretsky, Victor E. Alvarez, Brett Martin, Yorghos Tripodis, Joseph N. Palmisano, Raymond Nicks, Daniel A Kirsch, Thor D. Stein, Ann C. McKee

**Affiliations:** ^1^ Department of Neurology, Boston University Chobanian & Avedisian School of Medicine, Boston, MA, USA; ^2^ Boston University Alzheimer's Disease Research Center, Boston University Chobanian & Avedisian School of Medicine, Boston, MA, USA; ^3^ Boston University Chronic Traumatic Encephalopathy Center, Boston University Chobanian & Avedisian School of Medicine, Boston, MA, USA; ^4^ Boston University Chobanian and Avedisian School of Medicine, Boston, MA, USA; ^5^ Boston University Chronic Traumatic Encephalopathy Center, Boston, MA, USA; ^6^ Boston University Chobanian & Avedisian School of Medicine, Boston, MA, USA; ^7^ Alzheimer's Disease Research Center, Boston University Chobanian & Avedisian School of Medicine, Boston, MA, USA, Boston, MA, USA; ^8^ Boston University Alzheimer's Disease Research Center, Boston, MA, USA; ^9^ Department of Pathology and Laboratory Medicine, Boston University School of Medicine, Boston, MA, USA; ^10^ Department of Neurology, Boston University School of Medicine, Boston, MA, USA; ^11^ Department of Veterans Affairs Medical Center, Bedford, MA, USA; ^12^ VA Boston Healthcare System, Boston, MA, USA; ^13^ Boston University School of Public Health, Boston, MA, USA; ^14^ Boston University Chronic Traumatic Encephalopathy Chobanian and Avedisian School of Medicine, Boston, MA, USA; ^15^ Department of Pathology and Laboratory Medicine, Boston University Chobanian & Avedisian School of Medicine, Boston, MA, USA; ^16^ VA Bedford Healthcare System, Bedford, MA, USA

## Abstract

**Background:**

Repetitive head impacts from amateur contact sports can lead to structural brain injuries and long‐term neurodegeneration, including chronic traumatic encephalopathy (CTE). The goal of this study was to characterize the neuropathologic alterations and clinical symptoms of young amateur contact sport athletes.

**Method:**

This case series analyzes findings from 180 brain donors younger than 30 years, including 148 (82.2%) who played only at the amateur level, from the Understanding Neurologic Injury and Traumatic Encephalopathy (UNITE) Brain Bank. Neuropathologic evaluations, retrospective telephone clinical assessments, and online questionnaires with informants were performed blinded. Exposure was measured as years of play. Cognitive symptoms, mood disturbances, and neurobehavioral dysregulation were assessed using informant‐reported athletic history and informant‐completed scales.

**Result:**

Among 180 contact sports participants (mean [SD] age, 23.14 [4.32] yrs; 166 [92.2%] male), CTE was diagnosed in 70 (38.8%; median age, 25.3 (range 17‐29) yrs) who played American football, ice hockey, soccer, rugby, or wrestled. 148 were amateurs (82.2%) who played youth, high school or collegiate sports. Of the amateurs, CTE was diagnosed in 52 (35.1%) including one woman who played collegiate soccer. Most were diagnosed with mild CTE (stages I or II). Brain donors who had CTE were older (*p* <0.001, mean difference, 3.54 yrs; 95%CI, 2.44‐4.63 yrs). For those who played football, duration of playing career was significantly longer in those with CTE (*p* <0.001, mean difference, 3.45 yrs; 95%CI, 1.92‐4.98 years) (Table 1). Athletes with CTE had more ventricular dilatation, cavum septum pellucidum, and perivascular pigment‐laden macrophages in the frontal white matter than those without CTE. Interface astrocytosis and AQP4 alterations were found in a subset with CTE (Figure 1). Cognitive and neurobehavioral symptoms were frequent among all brain donors. Suicide was the most common cause of death; there were no differences in clinical symptoms or cause of death based on CTE status.

**Conclusion:**

Young amateur contact sports players are at risk for structural and microstructural brain injuries, including enlarged ventricles, cavum septum pellucidum, microvascular injury, blood‐brain barrier breach, interface astrocytosis, and glymphatic remodeling. Future studies are needed to clarify the association between these early structural injuries after RHI and risk for CTE.